# Toxicological Profile of Polyethylene Terephthalate (PET) Microplastic in Ingested *Drosophila melanogaster* (Oregon R^+^) and Its Adverse Effect on Behavior and Development

**DOI:** 10.3390/toxics11090782

**Published:** 2023-09-14

**Authors:** Simran Kauts, Yachana Mishra, Sumaira Yousuf, Rima Bhardwaj, Sandeep K. Singh, Fahad M. Alshabrmi, Mahmoud Abdurahman, Emanuel Vamanu, Mahendra P. Singh

**Affiliations:** 1 Department of Zoology, School of Bioengineering and Biosciences, Lovely Professional University, Jalandhar 144411, India; 2 Department of Chemistry Poona College, Savitribai Phule Pune University, Pune 411007, India; 3 Indian Scientific Education and Technology Foundation, Lucknow 226002, India; 4 Department of Medical Laboratories, College of Applied Medical Sciences, Qassim University, Buraydah 51452, Saudi Arabia; 5 Department of PH, College of Medicine, Imam Muhammad Ibn Saud Islamic University, Riyadh 13317, Saudi Arabia; 6 Faculty of Biotechnology, University of Agricultural Sciences and Veterinary Medicine, 011464 Bucharest, Romania; 7 Department of Zoology, Deen Dayal Upadhyaya Gorakhpur University, Gorakhpur 273009, India; 8 Centre of Genomics and Bioinformatics (CGB), Deen Dayal Upadhyaya Gorakhpur University, Gorakhpur 273009, India

**Keywords:** polyethylene terephthalate, microplastic, toxicity, *Drosophila*

## Abstract

Microplastics are readily available in the natural environment. Due to the pervasiveness of microplastic pollution, its effects on living organisms necessitate further investigation. The size, time of exposure, and amount of microplastic particles appear to be the most essential factor in determining their toxicological effects, either organismal or sub-organismal. For our research work, we preferred to work on a terrestrial model organism *Drosophila melanogaster* (Oregon R^+^). Therefore, in the present study, we characterized 2–100 µm size PET microplastic and confirmed its accumulation in *Drosophila*, which allowed us to proceed further in our research work. At larger dosages, research on locomotory activities such as climbing, jumping, and crawling indicated a decline in physiological and neuromuscular functions. Our studies also determined retarded development in flies and decreased survival rate in female flies after exposure to the highest concentration of microplastics. These experimental findings provide insight into the possible potential neurotoxic effects of microplastics and their detrimental effects on the development and growth of flies.

## 1. Introduction

Microplastics are pieces of plastic smaller than 5 mm in size [1]. Since the concept of microplastics was first proposed in 2004 by British scientists, there has been an increasing focus on this form of pollution. Prior research has shown that microplastics may be found in the air, water, and soil, as well as in seafood, salt, and edible items that we consume every day [2]. It has been shown via research that marine species are capable of ingesting microplastics, which may then cause bodily harm by obstructing digestive systems and feeding auxiliary organs [3]. In addition, microplastics have the potential to have toxicological effects, including oxidative damage, an increase in inflammation, a reduction in immunity, a problem with microorganisms that are present in the intestine, and hepatic metabolic abnormalities [4]. Both inhalation and ingestion are potential routes of microplastic exposure for humans.

A study [5] determined that the accumulation of polystyrene (PS) microplastics in the marine medaka (*Oryzias melastigma*) caused a delay in gonad maturation and a reduction in fecundity. Additionally, concentrations of 17β-estradiol (E2) were also found to be decreased in the female plasma. Furthermore, it was observed that the incubation period, egg hatching rate, heart rate, and body length of the offspring were also reduced. Furthermore, recent studies have shown that PS microplastics can induce apoptosis in different types of human cells, including monocytic leukemia cell lines (THP-1), colon carcinoma cells (Caco-2), and lung cancer cells (Calu-3) [6]. Due to the high surface area of microplastics, they contain organic contaminants and substances such as fluorobenzene, aromatic hydrocarbons, heavy metals, viruses, and bacteria, which introduce combined chemical pollution that can travel through the food chain and accumulate in living organisms, causing severe harm [7]. Currently, most of the attention paid to the dangers posed by microplastics is concentrated on either PS or polypropylene (PP) [8].

There have only been a few studies conducted regarding different types of microplastics. Due to this reason, for further investigation, polyethylene terephthalate (PET) was selected as the material to study based on different concentrations and durations of PET microplastic exposure. The primary use for PET is in the packaging industry, where it accounts for 71% of the overall consumption of plastic in Europe [9]. In addition, due to its strong resistance to impact, as well as its resistance to friction and its mechanical qualities, it is often used in the production of bottles for mineral water and carbonated beverages [10]. PET was found to make up 84% of reusable water bottles and 31% of beverage bottles [11]. As of April 2019, there were 7.7 billion people in the world (a staggering rise over previous estimates) and the quantity of garbage humans produce has increased along with the increase in population. Easy-to-dispose items such as cans and water bottles are necessary for people always on the move, but their usage has contributed to a worldwide increase in plastic pollution [12].

According to a study, microplastics have been spotted in human fecal matter, the most frequent of which are PP and PET [13]. At the same time, researchers discovered a total of 12 microplastic particles in the placentas of four different women [14]. The danger that PET microplastic poses to human life should not be disregarded, since this form of plastic is intimately connected to it. Despite the fact that in vivo research on mammals appears to be the most effective method for generating reliable data that can be used for risk assessment [15], these studies have significant drawbacks that may be attributed to ethical concerns and challenges in terms of manipulation, including increased expenses both monetarily and in terms of the amount of time required [16]. After comprehending the previously mentioned challenges, we selected a simplified animal model *Drosophila melanogaster* (Oregon R^+^) for assessing the hazards posed by microplastics by covering different parameters that were not discussed in the previous literature.

The fruit fly *Drosophila melanogaster* (Oregon R^+^) stands out among these other model species. This model offers a number of benefits for experimentation, including an abbreviated life cycle, straightforward manipulation, and ethical approval. In addition to this, their genome includes genes that are homologous to 75% of the genes implicated in human diseases [17]. Lately, *Drosophila* has been utilized for the purpose of assessing the possible dangers linked with being exposed to polystyrene nano- and microplastics [18]. We have investigated the potential risks of PET microplastic by determining its behavioral, developmental, and locomotory activities after ingestion of PET microplastic. Taking the benefits of *Drosophila* into consideration, we have been able to conduct this research.

## 2. Materials and Methods

### 2.1. PET Microplastic Formation

The PET plastic pellets were purchased from Sigma- Aldrich and then ground into a fine powder with the help of a grinder. PET plastic powder was then sieved with a 0.02 mm size sieve to obtain a 2–100 µm size PET.

### 2.2. PET Microplastic Characterization (FTIR, FE-SEM, and EDX)

For the FTIR (Fourier-transform infrared spectroscopy) study, PET microplastic was used as the sample for investigation. Sample discs with a clear appearance were made by carefully placing 10 mg of the material within a KBr pellet of 100 mg in weight. Then, an FTIR spectrophotometer from Perkin-Elmer was used to analyze the sample [19]. The FE-SEM (field emission scanning electron microscopy, JEOL model) image provided important information regarding the surface characteristics, structure, and dimensions of the particles. At various magnifications, PET microplastic was examined in this study. The EDX (energy dispersive X-ray spectroscopy) spectrum plainly displayed conspicuous particles, which allowed the composition and purity of the PET microplastic to be determined. The energy levels of these X-rays were then visualized as peaks in the spectrum, providing vital information regarding the composition and elemental makeup of the PET microplastic [20].

### 2.3. Examination of the Health Effects of Exposure to PET Microplastic Using Drosophila melanogaster (Oregon R^+^)

Under controlled conditions, *Drosophila melanogaster* (Oregon R^+^, wild-type strain) was fed with the conventional *Drosophila* diet of maize powder, agar, sodium benzoate, yeast, propionic acid, and sucrose [21]. The flies were maintained under specific environmental conditions, including a light–dark cycle of 12 h and a temperature of 24 ± 1 °C in a sterile laboratory setting at Lovely Professional University in Phagwara, Punjab, India.

### 2.4. Schedule for the Treatment of Drosophila

In the experimental design, third-stage *Drosophila melanogaster* larvae and adult flies were separated into five distinct groups. Group I served as the control and was cultured under *Drosophila* standard feed. Another group (Group II) was exposed to a mixture of ethanol and distilled water (vehicular control). Food containing PET was administered to Groups III, IV, and V containing concentrations according to the previous literature [22], with some modifications: 10 g/L, 20 g/L, and 40 g/L PET microplastic mixed with a 1:1 ratio of ethanol and distilled water (DW), respectively. Each group’s larvae were permitted to consume their respective food for 24 and 48 h. The flies were then subjected to a 15-day treatment to evaluate their behavioral and locomotory activity [23].

### 2.5. PET Microplastic Uptake by Drosophila melanogaster

**Figure 1:**
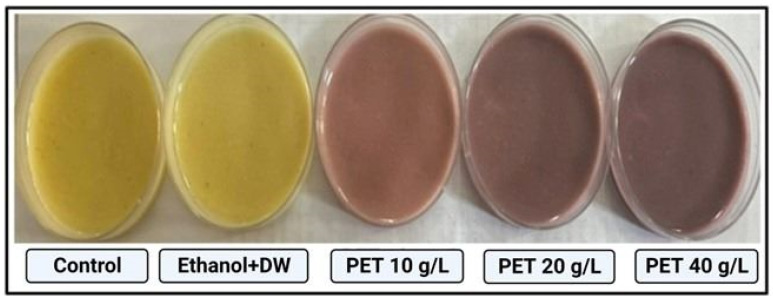
Demonstrating groups containing control diet and different concentrations of plastic-treated diet dyed with Nile red dye.

Accurate risk assessment in future investigations depends on identifying the accumulation of PET microplastic in *Drosophila.* A targeted approach was used to accomplish this objective. Staining with Nile red dye has become an easy-to-use low-cost method for assessing the detrimental impact on the environment of a broad variety of microplastics [24]. To stain the PET microplastic, the protocol was followed as mentioned by [19]. An amount of 1 mL of Nile red solution (0.50% in DMSO) was added. The stained pellet was washed several times using ethanol in a 0.10 M PBS solution at pH 7.40 to remove any remaining stain. Nile red-stained PET microplastic in the midgut and hindgut of *Drosophila* larvae is easily visible to the naked eye [25]. The accumulation of PET microplastic in the living tissue of groups treated with PET 10 g/L, 20 g/L, and 40 g/L concentrations of plastic is shown in Figure 1. The presence of microplastic was seen under a stereomicroscope and fluorescence microscope by observing dissected and whole larvae. This method provided light on the path taken by PET microplastic after ingestion.

### 2.6. Behavioural and Developmental Analysis

#### 2.6.1. Climbing Activity

According to a previous study, specific modifications were made to the ascent evaluation [26]. Twenty flies were placed in a plastic cylinder that was 20 cm in length and 2 cm in breadth. Any fly that made it over a 15 cm line after being lightly tapped at the bottom for 30 s was recorded, unless it had fallen into one of the vials. The percentage of inspected flies that were able to ascend 15 cm above the surface was the climbing count after 15 days of exposure. The data were shown as a percentage of the overall fly count (n^total^), which included the number of flies above (n^above^) and below (n^below^) 15 cm. Standard deviations were provided with reported findings based on counts from three independent assessments.

1/2[(n^tot^ + n^above^ − n^below^)/n^tot^]

#### 2.6.2. Jumping Activity

The activation of neuromuscular functions was evaluated using jumping activity [27]. It appeared that the frequency of locomotor activity influenced the jumping response threshold. To conduct the experiment, newly hatched flies were placed in a cylindrical container marked from 1 to 10 cm and their individual leap distances from the bottom of the container were measured. The average number of jumps performed over five repetitions was used to quantify the leaping behavior after 15 days of exposure. To ensure reliable results, each cohort of 100 flies was subjected to the experiment five times.

#### 2.6.3. Crawling Activity of Larvae

The larval crawling experiment was conducted using the protocol published in earlier papers. On a glass petri dish covered with 2% agarose, we placed nine third-instar larvae from the control and treatment groups that had been rinsed with PBS (pH 7.4) to eliminate any residues of food. Three separate 1-minute durations were observed of larvae crawling on an agar surface set on a graph sheet after 24 and 48 h of exposure. In order to determine how far each treatment group’s larvae traveled in 1 min, we measured how many grid lines in (cm) they traveled in 60 s and then determined the group’s mean [28].

#### 2.6.4. Emergence of Flies

Female flies were observed laying eggs synchronously for one hour, after which these eggs were collected on petri dishes containing regular food. After approximately 24 h of egg laying, newly emerged Oregon R^+^ strain first-instar larvae were transferred to various experimental groups. These groups included controls, a vehicular control, and three groups treated with 10 g/L, 20 g/L, and 40 g/L, respectively, of PET. Each group consisted of five replicates, each containing fifty larvae. On the first day, the first adult fly emerged and continued until each and every fly had been enclosed in the control group; the total number of flies emerging from each group was counted and then compared to the control. Using the methodology described previously with small modifications, the development of the flies in each of the categories was assessed [29,30].

#### 2.6.5. Survival Assay

The effects of prolonged exposure of *Drosophila* to PET microplastic were evaluated using a modified version of a previously established survival experiment [31,32]. The lifespans of 50 adult male and female flies were separately monitored as they were fed from vials containing control, ethanol + DW, and PET microplastic feed (10, 20, or 40 g/L) until their deaths. Vials of food were stored horizontally and replenished every week. The number of dead flies in the food vials was counted every day, excluding flies that had fled or been adhered to the food. After the flies had all perished, a survival curve was produced using GraphPad Prism 6. Then, the log-rank Mantel–Cox test was run to determine statistically significant differences in outcomes between the various treatment groups.

#### 2.6.6. Statistical Analysis

In our research work for statistical analysis, we used one-way ANOVA, Tukey’s comparison test, and the log-rank Mantel–Cox test to identify the significant difference in the results.

## 3. Results

### 3.1. Characteristics’ Analysis of PET Microplastic

#### 3.1.1. FTIR

**Figure 2:**
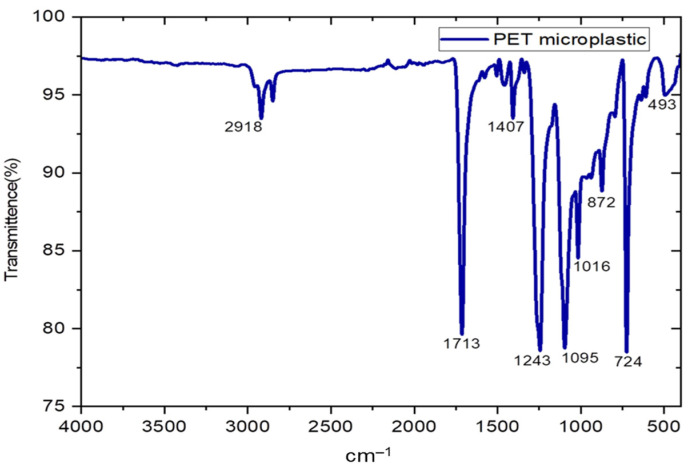
FTIR analysis of polyethylene terephthalate microplastic.

The FTIR analysis of PET microplastic revealed a number of distinct bands with distinct peak wavenumbers in cm^−1^. For example, the peak at 2918.51 cm^−1^ corresponded to CH stretching, whereas the band at 1713.39 cm^−1^ indicated C=O stretching of the ester group. Other notable peaks included 1407.25 cm^−1^, which indicated the presence of the benzyl ring, and 1243.9 cm^−1^, which was ascribed to the elongation of the ester C=O bond. In addition, vibrations of the ester group were detected at 1095.88 cm^−1,^ and benzene’s in-plane vibration was detected at 1016.02 cm^−1^. The peaks at 872.58 cm^−1^ and 724.43 cm^−1^ corresponded, respectively, to the swaying of glycol and the out-of-plane benzene group. Lastly, the peak at 493.97 cm^−1^ indicated the out-of-plane vibration of =C-H in the benzene group. These FTIR results, as shown in Figure 2, verified that PET plastic is composed of repeating units of the monomer ethylene terephthalate; it is important to note that ester carbonyls are typically found in the range of 1710–1725 cm^−1^ [25].

#### 3.1.2. SEM and EDS

**Figure 3:**
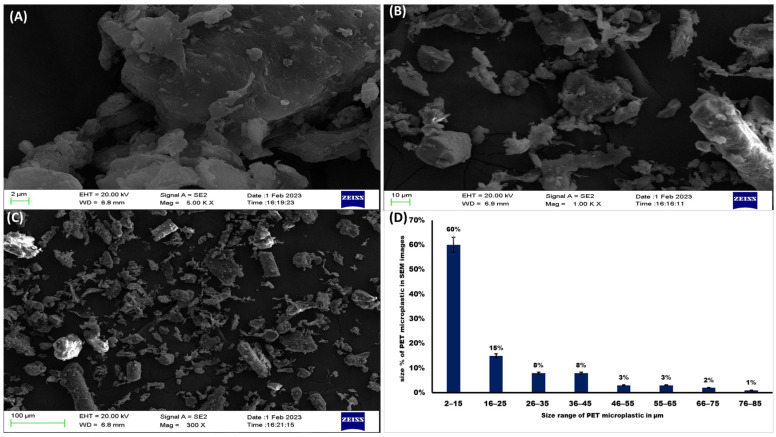
SEM-EDS analysis of PET microplastic. Images (**A**–**C**) of the PET microplastic demonstrated that particles are irregular in shape and sizes vary from 2 to 100 µm determined by Image J software. (**D**) Size % of PET microplastic in SEM.

**Figure 4:**
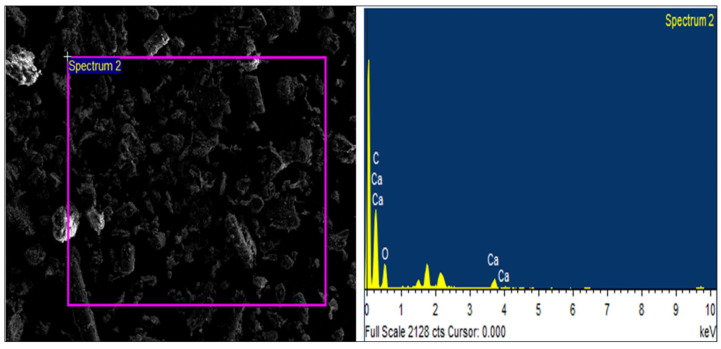
EDX confirmed the PET’s elemental composition, indicating that it contains 53.65% carbon, 42.42% oxygen, and 3.93% wollastonite.

SEM is extensively utilized for assessing the size and morphology of PET microplastic due to its ability to provide high-resolution images. The SEM images obtained displayed the irregular shapes of PET microplastic, which included cylindrical, oval, circular, triangular, and even some with indistinct shapes. The SEM images in Figure 3A–C depict the various shapes and sizes observed. We determined the size % of PET microplastic in the SEM images by Image J software (version 1.53), as shown in Figure 3D. Additionally, Figure 4 presents the EDS analysis, revealing the elemental composition of the PET microplastic.

### 3.2. PET Microplastic Accumulation

**Figure 5:**
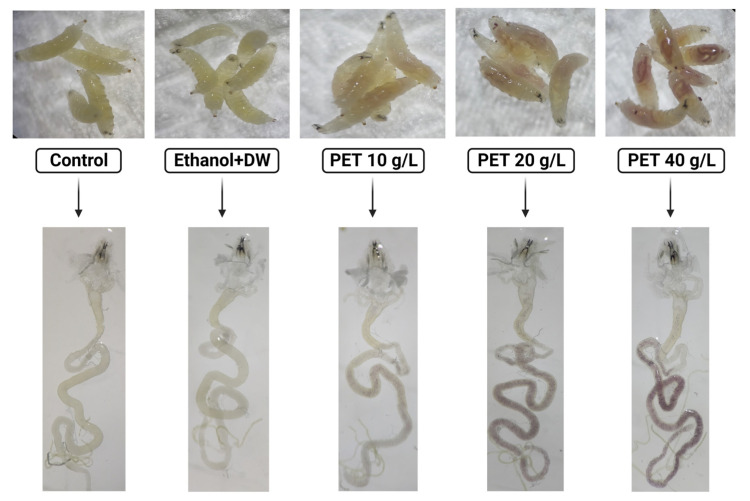
Accumulation of PET microplastic in the midgut and hindgut of the whole and dissected larvae in the groups (control, ethanol + DW, PET 10 g/L, PET 20 g/L, and PET 40 g/L, respectively). Plastic dyed with Nile red dye is clearly visible due to the translucent and membranous texture of the *Drosophila* larvae cuticle.

**Figure 6:**
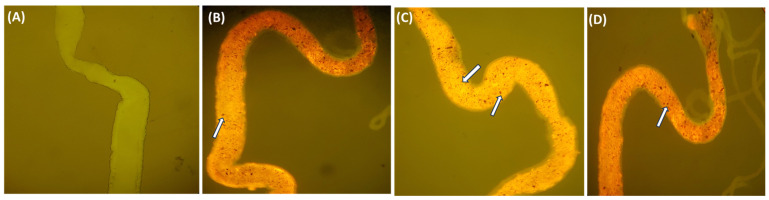
Showing accumulation of PET microplastic. (**A**) *Drosophila* larvae gut does not contain PET microplastic; (**B**,**C**) midgut with Nile red-stained PET microplastic; (**D**) hindgut with Nile red-stained microplastic. The presence of microplastic is represented by white arrows.

Nile red dye is the most suitable stain for microplastic identification and detection due to its strong adsorption for plastics, enhanced fluorescence intensity, faster incubation time, and excellent affinity for a broad variety of plastic polymers. The results indicate the accumulation of PET microplastic according to the concentrations in the midgut and hindgut of the larvae under the stereomicroscope shown in (Figure 5) using a (Labomed Lx 400 eFL2, Labomed India, Gurgaon, India) fluorescence microscope. The fluorescence intensities of Nile red-stained PET microplastic were observed when excited within the range 440–520 nm and emission within the range 445–545 nm at 10× magnification [33], as shown in (Figure 6). This analysis provides a platform to ensure the accumulation of PET microplastic, which helps in further investigation.

### 3.3. Behavioral and Developmental Effects

#### 3.3.1. Effect of PET Microplastic on (Locomotion) Climbing Activity of Drosophila

**Figure 7:**
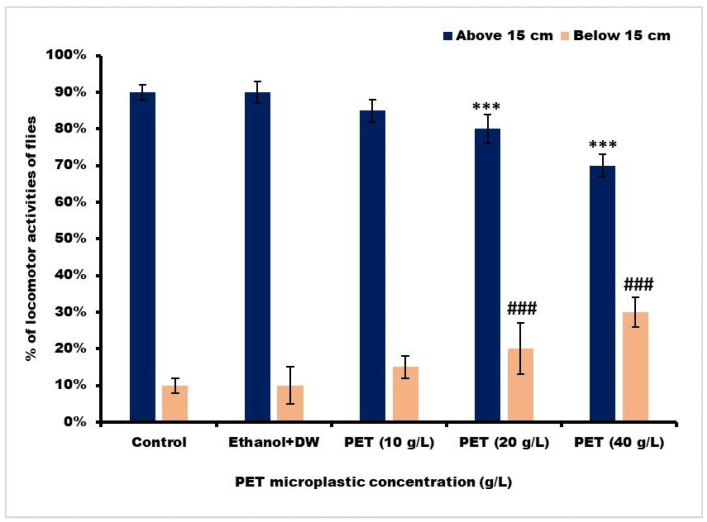
The climbing activity of *D. melanogaster* (Oregon R^+^) flies was assessed after a 15-day exposure period. Significance with mean ± SEM was denoted as *** for *p* < 0.001 in comparison with Groups I and II above 15 cm. Similarly, significance was denoted as ### for *p* < 0.001 in comparison with Group I below 15 cm. In this context, DW refers to distilled water and PET represents polyethylene terephthalate.

The control and ethanol + DW-treated flies showed their full capacity for climbing after 30 s (only a 10% decrease). As demonstrated in Figure 7, the climbing ability of the PET 20 g/L and PET 40 g/L groups significantly decreased, making it challenging for them to scale the plastic tube walls. However, there was no noticeable difference in the PET 10 g/L group; significance was denoted by using one-way ANOVA and Tukey’s comparison test.

#### 3.3.2. Effect on the Jumping Activity of Flies after Accumulation of PET Microplastic

**Figure 8:**
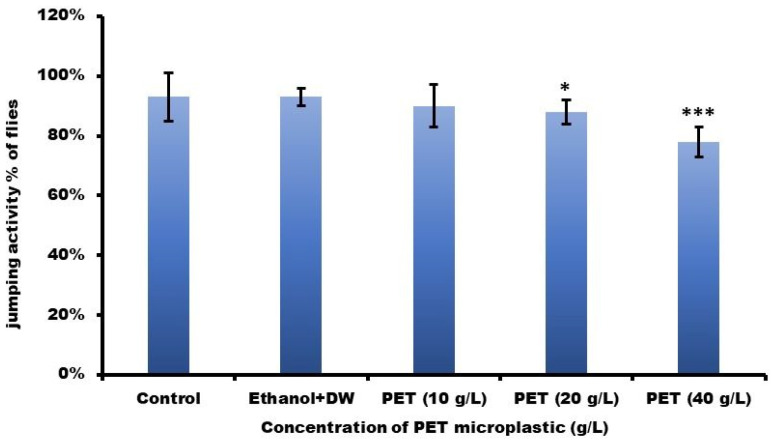
The jumping activity of *D. melanogaster* (Oregon R^+^) flies was examined after a 15-day exposure period. Significance was denoted as *** *p* < 0.001 and * *p* < 0.5 vs. Group I with mean ± SEM. In this context, DW refers to distilled water and PET represents polyethylene terephthalate.

Flies exposed to PET 20 g/L and 40 g/L showed significantly less jumping behavior, which decreased by 5 and 15%, respectively, compared with those exposed to the control and ethanol + DW (5%). As shown in Figure 8, the jumping ability of flies treated with 10 g/L with PET did not significantly decline. The mean ± SEM was compared using one-way ANOVA and Tukey’s comparison test. At *** *p* < 0.001, statistical significance was indicated.

#### 3.3.3. Crawling Ability of Drosophila Larvae

**Figure 9:**
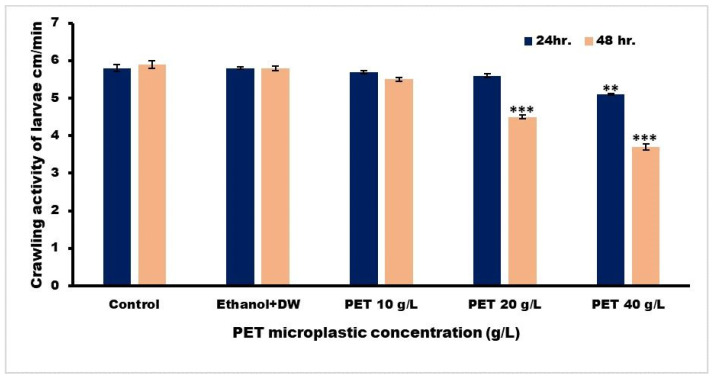
Graphical illustration of the path traveled by third-instar larvae in one minute. The statistical significance was determined as *** *p* < 0.001 and ** *p* < 0.01 vs. Group I.

*Drosophila* larvae may be tested for the health of their ventral ganglia motor neurons by observing how well they move. As the larvae move by contracting their body wall musculature, any impairment in this locomotor function can be indicative of neuronal damage [32]. To determine whether microplastic is hazardous to *Drosophila*, researchers may apply the larval crawling test, which measures how much it hinders the larva’s ability to move about on its own. The average rates of travel in Groups IV and V showed slow crawling activity, as shown in Figure 9. The mean ± SEM was compared using one-way ANOVA and Tukey’s comparison test.

#### 3.3.4. Emergence of Flies

**Figure 10:**
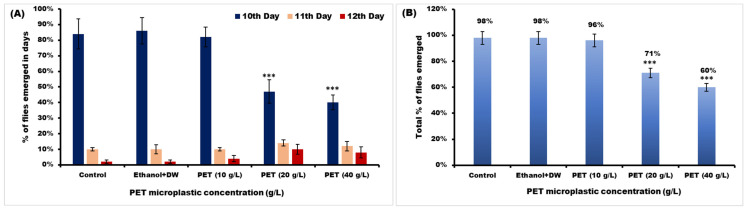
Emergence % of flies after being exposed to PET microplastic. (**A**) The emergence percentages of flies were recorded for 3 consecutive days, specifically on the 10th, 11th, and 12th days. Statistical significance with mean ± SEM was denoted as *** *p* < 0.001 when compared to Group I. (**B**) The total percentage of emerged flies across all groups up to 12 days was calculated. In this context, DW refers to distilled water and PET represents polyethylene terephthalate.

In the control group, the number of flies emerged by 98%, and in the ethanol+DW (vehicular control) group, flies emerged in 12 days. In PET (10 g/L), PET (20 g/L), and PET (40 g/L), flies emerged by 94%, 71%, and 60%, respectively, as shown in Figure 10. Some of the flies were left to emerge in groups PET (20 g/L) and PET (40 g/L). Only the mentioned percentage of flies managed to emerge in 12 days. The mean ± SEM was compared using one-way ANOVA and Tukey’s comparison test.

#### 3.3.5. Survival Assay

**Figure 11:**
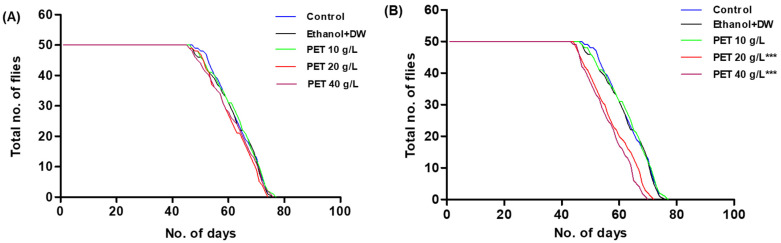
(**A**) The survival rate of male flies does not show any significant difference in the life span of the PET microplastic-containing group vs. control. (**B**) Showing a significant decrease in the life span of female flies in Groups IV and V, denoted as *** *p* < 0.001, in comparison with the control group.

The long-term toxicity of PET microplastic was tested by a survival assay. There was no statistically significant difference in the survival of the male flies, as shown in (Figure 11A), but there was an observed decrease in the life span of female flies in PET 20 g/L and 40 g/L, as shown in (Figure 11B), according to statistical analysis from the log-rank Mantel–Cox test. The median survival rate of male flies in the control group, ethanol +DW, PET 10 g/L, PET 20 g/L, and PET 40 g/L was 47.5, 46, 46, 47, and 47 days and for female flies was 48, 47,47, 43, and 41 days, respectively.

## 4. Discussion

**Figure 12:**
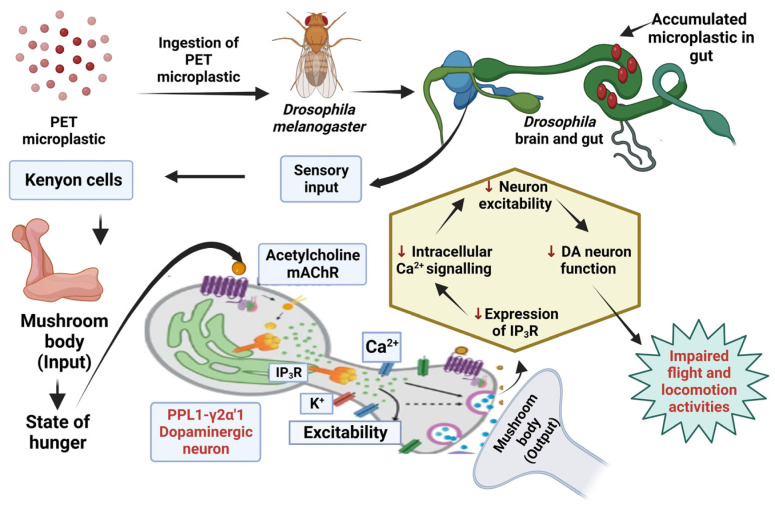
Schematic illustration of PET microplastic accumulation in drosophila and its possible effects on the function of dopaminergic neurons, which could be responsible for fly behavior and locomotion. DA—dopaminergic, Ca—calcium ions, K—potassium ions.

To investigate the detrimental effects of PET microplastics on brain-related activities, studies were conducted using pure PET samples. Characterizing the size, shape, elements, and chemical structure of PET microplastics is crucial for understanding their properties. FTIR technique was employed to analyze the characteristics of PET microplastic and to identify functional groups present. We also explored the catalytic interaction between enzymes and substrates, as well as the validation of bioactive substances covalently bound to microplastics. Our FTIR analysis, supported by a previous study [25], confirmed the affiliation of these particles with PET. SEM and EDS analyses were conducted to examine the morphological texture and element configuration of the PET microplastic. SEM revealed irregular particles of microplastics with varying diameters, with some particles observed adhering to one another. The size of the particles was measured using ImageJ software and was found to be between 2 and 100 µm. EDS analysis provided an elemental composition, indicating that PET microplastics contain 53.65% carbon, 42.42% oxygen, and 3.93% wollastonite. Considering ingestion as the primary exposure route for microplastics, we conducted further experiments after determining their accumulation in the body. Our study focused on the behavioral activity of *Drosophila* after exposure to PET microplastics with different concentrations and durations. Behavior can shed light on an organism’s physiological processes [27] and, in the case of *Drosophila,* their climbing and jumping abilities provided insights into their overall health. We observed significant impairments in locomotor activity in *Drosophila* exposed to PET microplastics at concentrations of 20 g/L and 40 g/L. These impairments were characterized by an inability to synchronize leg motions, resulting in the insects being stuck at the bottom of the plastic cylinder. The observed locomotor deficits could also be associated with higher energy demands in mitochondria-rich muscles required for locomotion and flight. Uncoupled mitochondrial mechanisms and severe complex I inhibition have been linked to such locomotor difficulties, which could also be a reason for behavior deformities [34]. Our results also supported previous studies [35] that have associated dopamine deficits with mobility disabilities, because dopaminergic neurons play a vital role in the behavior and locomotion of flies, as shown in Figure 12. This schematic representation suggests that a disruption in the neurons may be the cause of their impaired locomotion. It is necessary to conduct additional research to determine whether the accumulation of PET microplastics can disturb dopaminergic neurons, thereby altering the physiological processes of flies. Another study, which was conducted on zebrafish, also stated that the functioning of dopaminergic neurons was associated with behavior and locomotion activities [36].

Similar to the findings in other organisms, our study revealed a decrease in crawling speed in *Drosophila* larvae exposed to PET microplastic compared with their normal crawling speed after being exposed to high concentrations of PET microplastic. The previous literature [37] has observed decreased swimming activity in *Sebastes schlegeli,* which supported our study. Moreover, exposure to microplastics in *C.elegans* has been shown to inhibit acetylcholinesterase function and alter neurotransmitter levels, leading to behavioral abnormalities [38]. In addition to locomotor impairments, we observed a delayed development, which refers to the decrease in the emergence percentage of *Drosophila* in groups after being exposed to high doses. This decrease could be attributed to the disruption of vital processes during early development caused by exposure to reactive oxygen species (ROS) [39]. ROS exposure during organogenesis can lead to delayed or abnormal development and can subsequently delay emergence [40]. However, the effects of ROS on development are complex and context-dependent. While excessive ROS levels can be harmful, they also play a role in signaling processes that regulate normal development [41]. The timing of emergence may be determined by the delicate balance between ROS-mediated signaling and oxidative stress [42]. In order to support this study, the previous literature [43,44] has also stated that exposure to polystyrene microplastic copepods and brown shrimps showed a delay in development. Our study demonstrated that the life span of female *Drosophila* decreased after the consumption of high concentrations of PET microplastic, but for male flies there was no effect on life span. Another study [21] also observed the changes in the life span of *Drosophila* after consumption of microplastic. The previous literature has shown that the mortality of the marine species *Clarias gariepinus* (African catfish) increased by 10% after being exposed to a high concentration (2 g/L) [45]. Our findings have provided preliminary information for future research on the impact of PET microplastic on *Drosophila*. The impaired locomotion and negative effect on development and life span following plastic consumption could be related to neurological signaling defects and genetic conditions such as maternal effect, changes in segmentation, or homeotic gene; therefore, further research is required regarding molecular and cellular aspects in order to elaborate on the reason for these challenges considering the findings of this research [46].

## 5. Conclusions

Overall, our findings suggest that toxicity is concentration- and time-dependent: the higher the plastic concentration, the greater the toxicological effect. At higher dosages, the behavior, locomotion, development, and life span (female) of flies are adversely affected. Concerning the population, our findings indicate that a certain concentration of plastic is acceptable for use. However, because plastic has become an integral part of our daily lives, we cannot entirely disregard it. However, if the concentration exceeds a particular threshold, this is cause for grave concern. The underlying toxicity discussed in this study may indicate that plastic is having a gradual and constant effect on our lives. These results shed light on the severe repercussions of microplastic pollution and highlight the imperative need for effective mitigation strategies. Therefore, additional research is required to evaluate the effects of microplastic on neurological aspects and to identify the molecular or cellular cause of *Drosophila’s* delayed development and growth after microplastic ingestion.

## Data Availability

Data are contained within the article.
